# Hand hygiene and HAI transmission in Piedmont, Italy: an observational study, 2017-2019

**DOI:** 10.1093/eurpub/ckac130.231

**Published:** 2022-10-25

**Authors:** A Russotto, C Vicentini, CM Zotti

**Affiliations:** Department of Public Health and Paediatrics, University of Turin, Turin, Italy

## Abstract

**Background:**

Hand hygiene (HH) is one of the most important measures to prevent healthcare-associated infections (HAIs). Several indicators have been proposed to measure HH practices, including alcohol-based handrub consumption (AHC). The objective of this study was to evaluate whether AHC is associated with HAI transmission.

**Methods:**

All 25 public hospitals/trusts of Piedmont, in Northern Italy, are required to provide data each year concerning HAIs and infection prevention and control (IPC) activities, as part of a mandatory regional indicator system. Data on AHC and HAIs were extracted from reports provided concerning the years 2017-2019. The mean annual AHC for each facility was expressed as liters per patient-day. The rate of hospital-wide Methicillin-resistant Staphylococcus aureus (MRSA) and carbapenem-resistant Enterobacteriaceae (CRE) infections was calculated per 1000 patient-days (pd). Mean ventilation-associated infections (VAP) for 1000 days of ventilation (vd) and mean catheter-related bloodstream infections (CR-BSI) for 1000 days of catheterization (cd) were calculated for intensive care units (ICUs). Spearman's correlational analysis was conducted between AHC and HAIs: hospital-level, ICU-level.

**Results:**

Hospital-level: mean AHC was 15.4 liters/pd; mean HAI rate was 39.6/1000 pd (MRSA) and 18.5/1000 pd (CRE). No significant correlation was found. ICU-level: mean AHC was 39.6 liters/pd; mean HAI rate was 1.7/1000 cd (CR-BSI) and 8.2/1000 vd (VAP). A moderate correlation was found between AHC and CR-BSI rate (Spearman's ρ -0.55, p 0.022). Concerning AHC and VAP, higher AHC was reported from facilities with lower VAP rates, however no significant correlation was found (Spearman's ρ -0.203, p 0.451).

**Conclusions:**

Results of this study support the validity of AHC as an indicator of IPC practices in ICUs. The hospital-level analysis could have been affected by important differences in AHC among ward types.

**Key messages:**

- Results of this study support the association between higher AHC and reduced HAI transmission in intensive care units.
- Stratifying data by ward type could provide further insight in the association between AHC and HAI rates.

